# Evaluating the effect of lactic acid bacteria fermentation on quality, aroma, and metabolites of chickpea milk

**DOI:** 10.3389/fnut.2022.1069714

**Published:** 2022-12-05

**Authors:** Panling Zhang, Fengxian Tang, Wenchao Cai, Xinxin Zhao, Chunhui Shan

**Affiliations:** School of Food Science, Shihezi University, Shihezi, China

**Keywords:** lactic acid bacteria fermentation, soymilk functional properties, soymilk antioxidants, soymilk flavors, soymilk metabolites

## Abstract

Legumes are an attractive choice for developing new products since their health benefits. Fermentation can effectively improve the quality of soymilk. This study evaluated the impact of *Lactobacillus plantarum* fermentation on the physicochemical parameters, vitamins, organic acids, aroma substances, and metabolites of chickpea milk. The lactic acid bacteria (LAB) fermentation improved the color, antioxidant properties, total phenolic content, total flavonoid content, lactic acid content, and vitamin B6 content of raw juice. In total, 77 aroma substances were identified in chickpea milk by headspace solid-phase microextraction with gas chromatography/mass spectrometry (HS-SPME-GC-MS); 43 of the 77 aroma substances increased after the LAB fermentation with a significant decrease in beany flavor content (*p* < 0.05), improving the flavor of the soymilk product. Also, a total of 218 metabolites were determined in chickpea milk using non-targeted metabolomics techniques, including 51 differentially metabolites (28 up-regulated and 23 down-regulated; *p* < 0.05). These metabolites participated in multiple metabolic pathways during the LAB fermentation, ultimately improving the functional and antioxidant properties of fermented soymilk. Overall, LAB fermentation can improve the flavor, nutritional, and functional value of chickpea milk accelerating its consumer acceptance and development as an animal milk alternative.

## Introduction

With growing health awareness, consumers are now selecting healthy food products, such as soymilk enjoyed widespread interest (1, 2). Soy grains are rich in nutrients such as proteins, vitamins, and minerals, which can be extracted in water as soymilk. Also, soymilk is a preferred choice for those who are lactose intolerant, allergic to milk protein, or vegetarian (3). And soymilk is an ideal food to prevent some diseases, such as high blood pressure, high blood cholesterol. Moreover, Soybean and soy products are rich in nutrients, especially fermented soy products (fermented soymilk), which have health benefits such as: antioxidant effect, anticancer effect, and anti-inflammatory effect due to the presence of bioactive substances (4–6). And fermented soy products can also used as potential source of functional foods and bioactive peptides for developing nutritional products (7). Lactic acid bacteria (LAB) fermentation can improve the nutritional and functional values of soymilk (8). *Lactobacillus plantarum* bacteria grow well in soymilk, produce some bioactive peptides and reduce soy oligosaccharides in a strain-specific manner (9). *Lactobacillus plantarum* fermentation improves the antioxidant activity of soymilk food products (10). The soymilk fermented with *L. plantarum* was also found to lower the concentration of total cholesterol, triglyceride and low-density lipoprotein cholesterol in serum (11). And, compared to fresh soymilk, the concentrations of the characteristic flavor compounds for fermented soymilk using *L. plantarum* increased, while the contents of beany substances were decreased like hexanal, 2-pentylfuran, and 2-pentanone (12).

Although the effect of LAB fermentation on soybean products has been extensively investigated, little is known about the same for chickpeas, which can be a potential substitute of soymilk for its nutritional and organoleptic properties (2). Chickpeas (*Cicer arietinum L.*), one of the oldest and most widely consumed beans, are enriched in proteins (21–25%), fiber, and minerals. Also, chickpeas have high levels of resistant starch and amylose, which may reduce the onset of type II diabetes and hypertension. Chickpea milk is often regarded as an attractive milk substitute (13, 14).

Food flavor is one of the most decisive features of consumer acceptance (15, 16). Headspace solid-phase microextraction with gas chromatography/mass spectrometry (HS-SPME-GC-MS) has been widely adopted in environment samples, biology samples, especially food samples for odor analysis, quality classification, pesticide residue determination (17, 18). The displeasing beany flavor of soy products limits their consumption (9). The lactic acid bacteria fermentation significantly reduces the beany flavor of soymilk. Moreover, LAB fermentation can provide characteristic flavor to fermented soymilk by producing active reductases and unique flavor substances (19). Furthermore, LAB fermentation can convert certain off-favor aldehydes into corresponding alcohols and acids with fruity and sweet notes (20).

Lactic acid bacteria fermentation involves complex chemistry modulating the quality attributes of fermented foods by producing small molecule metabolites, which can be possibly tailored to achieve specific nutrition and flavor needs (21). Metabolomics, with high objectivity and reliability, offers a comprehensive and quantitative overview of metabolites in biological systems (22). Metabolomics is often used to assess critical metabolites related to food quality (23). Untargeted metabolomics examines a wide range of metabolites in a sample without a previous understanding and can also be used to describe the metabolism of a whole microorganism (24). Therefore, the same method can be used to determine the fermentation characteristics of chickpea milk.

So far, although many studies have examined the functional properties of fermented soy foods, the systematic descriptions of their quality, flavor, and metabolites are scarce, especially of the fermented chickpea milk. *Lactobacillus plantarum* can better adapt to growth-limiting substrates such as organic acids, polyphenols, etc. Therefore, *L. plantarum* fermentation can be a good choice for chickpea milk. Here, we investigated the effect of *L. plantarum* fermentation on physicochemical parameters, cell viability, and antioxidant properties of chickpea milk. Also, variations in organic acid and vitamins were determined by HPLC. The post-fermentation changes in flavor substances and metabolites were analyzed by headspace solid-phase microextraction with gas chromatography/mass spectrometry (HS-SPME-GC-MS) as well as untargeted metabolomics analysis via ultra high performance liquid chromatography/mass spectrometry (UHPLC-MS). Our results uncover the qualitative changes in fermented chickpea milk, which may be used to study its functional properties in the future.

## Materials and methods

### Preparation of chickpea milk

Chickpeas (Shihezi Market, Xinjiang, China) were soaked overnight in water (1:5 bean: water, m/v). The next day, removing moldy and insect-infested chickpeas, one part of the soaked beans was crushed in six parts of water (2) with heat processing for 30 min by mixer grinder (Joyoung, JYL-Y921, China). Then the soy drink was separated from solid residue using sterile gauze, eliminating impurities to make soymilk with fine taste and homogeneous texture. About 250 mL of soymilk sample was prepared by boiling for 10 min to confirm pasteurization.

### Fermentation of chickpea milk

The commercial strain *L. plantarum* (LP-56) was obtained from CINOBIO-TEC Co., Ltd. (Shanghai, China), and preserved at −20°C. The lyophilized strain powder was dissolved in sterile distilled water (1:50, w/v) (25), which was used to inoculate (0.07%, w/v) chickpea milk at 10 log CFU/mL (cell count at activation), as determined by spread plate method. The fermentation was performed for 24 h at 37°C (9). Samples were collected in triplicates at 0 and 24 h.

### Determination of physicochemical indices

In total, 1 mL of sample was added with 9 mL of saline (0.9%, w/v) and then serially diluted with saline multiple times in the same ratio. 1 mL of respective dilution was plated onto a Petri dish containing about 15 mL of the MRS agar medium. The inoculated Petri dish was incubated at 37°C for 48 h and the bacterial count was estimated as described previously (26).


(1) $$\text{N}=\frac{\sum \text{C}}{\left(\text{n}\,1+0.1\,\mathrm{n2}\right)\,\text{d}}$$

Where *N* = the number of microorganisms in the sample, ΣC = microbial total number, n_1_ = the number of plates for the first dilution; n_2_ = the number of plates for the second dilution; d = dilution factor.

The β-glucosidase activity was determined as described previously with some modifications (27). Took 0.1 mL of fermented soymilk, added 0.2 mL of sodium phosphate buffer (0.1 mol/L, pH 7.0) containing 5 mM *p*-NPG (*p*-nitrophenyl-β-D-glucoside) and put it into a 37°C water bath for 30 min. Then 0.5 mL of 1 mol/L NaCO_3_ solution (4°C) was added to terminate the reaction, followed by centrifugal treatment (8,000 r/min, 30 min, 4°C), and the supernatant was taken to determine the absorbance value at 400 nm. Control group was inactivated with boiling water for 5 min instead of heating with water bath 37°C for 30 min. One unit of β-glucosidase activity is defined as the amount of enzyme required to catalyze the formation of 1 μmol of ρ-nitrophenol per minute under the assay conditions. Standard curve: y  =  0.1028x  -   0.0078, r^2^ =  0.9999.

The sample color evaluation was performed with a colorimeter to obtain the L*, a*, and b* values.

The total protein and total fat contents were determined spectrophotometrically following the Chinese National Standard GB5009.5-2016 and GB5009.6-2016, respectively. The method details are mentioned in Supplementary Text 1.

### Determination of functional components

The total phenolic content (TPC) was determined using the Folin-Ciocalteu colorimetric method by measuring absorbance at 765 nm (26). This value denotes mg gallic acid equivalent (GAE) per mL of soymilk. The total flavonoid content (TFC) was determined using the aluminum nitrate colorimetric method by measuring absorbance at 508 nm (28). This value denotes mg rutin equivalent (RE) per mL of soymilk. The method details are in Supplementary Text 2.

### Determination of antioxidant activity

The DPPH (2,2-diphenyl-1-picrylhydrazyl) radical cation (DPPH^+^) scavenging activity of the samples was estimated spectrophotometrically at 517 nm as described previously with some modifications (29). The control group used methanol in place of the DPPH^+^ solution, while the blank samples had deionized water. The calculation was performed as follows:


(2) $${\text{DPPH}}^{+}radicalscavengingrate\left(\%\right)=\frac{\text{A}\,2-\mathrm{A1}}{\text{A}\,0}\times 100\%$$

Where A_2_, A_1_, and A_0_ are the absorbance of experimental, control, and blank samples, respectively.

The ABTS [2,2′-azino-bis(3-ethyl-benzothiazoline)-6-sulphonic acid] radical cation (ABTS^+^) scavenging activity of the samples was estimated by spectrophotometry at 734 nm as reported previously with some modifications (30). In control samples, absolute ethanol replaced the ABTS^+^ solution. The hydroxyl radical cation (OH^–^) scavenging activity was analyzed spectrophotometrically at 510 nm (31). In control samples, deionized water replaced the ethanol-salicylic acid, FeSO_4_, and H_2_O_2_ solutions, while the blank samples had deionized water. The calculation was performed as follows:


(3) $$Radicalscavengingrate\left(\%\right)=\left(1-\frac{\text{A}\,2-\mathrm{A1}}{\text{A}\,0}\right)\times 100\%$$

Where A_2_, A_1_, and A_0_ are the absorbance of experimental, control, and blank samples, respectively.

### Determination of vitamin B5 and B6 contents

Following the China National Standard GB5009.210-2016, vitamin B5 content was estimated by high performance liquid chromatography (HPLC) equipped with an ultraviolet spectrophotometric detector (Shimadzu, LC-2010, Japan). The HPLC conditions were as follows: chromatographic column, C18 column (4.6 mm × 250 mm, 5 μm); mobile phase, potassium dihydrogen phosphate solution (0.02 mol/L): acetonitrile (95:5); flow rate, 1 mL/min; column temperature, 30°C; detection wavelength, 210 nm; injection volume, 20 μL. The vitamin B5 standard solutions were 0, 2, 4, 8, 16, and 32 μg/mL (y = 106295x – 13160, r^2^ = 0.998).

Following the China National Standard GB5009.154-2016, vitamin B6 content was also estimated by HPLC equipped with a fluorescence detector (Shimadzu, LC-2010, Japan) under the following conditions: chromatographic column: C18 column (4.6 mm × 250 mm, 5 μm); mobile phase: methanol 50 mL, sodium octane sulfonate 2 g, and triethylamine 2.5 mL were dissolved in 1,000 mL ultrapure water of pH 3.0 ± 0.1 adjusted with glacial acetic acid; detection wavelength: excitation 293 nm, emission 395 nm; injection volume: 20 μL. The vitamin B6 standard solutions were 0, 0.1, 0.2, 0.4, 0.6, and 1.0 μg/mL (y = 6000000x – 154981, r^2^ = 0.995).

### Determination of lactic and citric acid contents

Analysis of organic acids was performed as follows (32): the sample was centrifuged and the obtained supernatant was filtered using a 0.45 μm membrane filter. 20 μL of the filtered sample was injected into a Waters 2998 HPLC system with a C18 column (4.6 mm × 250 mm, 5 μm) and diode-array detector (DAD). The conditions were as follows: mobile phase, 0.1% phosphoric acid + methanol (97 + 3, v/v); elution, isometric; column temperature, 40°C; detection wavelength, 210 nm. The standard solutions were 0, 4, 10, 20, 40, 100, 200, and 400 μg/mL (lactic acid: y =  243.07x + 163.46, r^2^ =  0.999; citric acid: y = 591.87x + 170.28, r^2^ = 0.999).

### Headspace solid-phase microextraction with gas chromatography-mass spectrometry analysis

In total, 5 mL of the sample was placed in a 20 mL headspace vial and equilibrated on a magnetic stirrer for 20 min at 60°C. An SPME fiber 50/30 μm carboxyene/dimethicone/divinylbenzene (CAR/PDMS/DVB) was extended through the needle and exposed in the vial and adsorbed in the headspace for 30 min. Afterward, the fiber was immediately injected into the GC inlet and desorbed at 250°C for 3 min. The samples were measured by GC-MS (Agilent 8890-7000D, USA) and the analysis of volatile compounds was performed in the splitless injection mode on an Agilent 19091N-133 with an HP-INNO Wax column (30 m × 0.25 mm, 0.25 μm). High-purity helium was the carrier gas at a constant flow rate of 1 mL/min. The GC oven was set to 40°C for 3 min, ramped to 82°C at 2°C/min, then to 103°C at 1°C/min, 124°C at 7°C/min, 138°C at 2°C/min, and finally 220°C at 10°C/min to hold for 3 min. Qualitative analysis of volatile compounds was identified by matching the National Institute of Standards and Technology database (NIST17.L) and retention index (RI).

### UHPLC/MS analysis

Analysis of metabolites by UHPLC/MS (Thermo Scientific, Thermo Fisher Scientific Inc., Waltham, MA, USA) according to the previous method with some modifications (33). Sample preparation: 20 mg sample was extracted with 400 μL of acetonitrile: methanol solution (1:1, v:v) containing 0.02 mg/mL of L-2-chlorophenylalanine (internal standard); Frozen tissue was ground for 6 min at −10°C and 50 Hz; Cryogenic ultrasonic extraction was performed for 30 min at 5°C and 40 KHz; The samples were incubated at −20°C for 30 min and then centrifuged for 15 min at 13.000 × *g* and 4°C, Also, 20 μL of supernatant from each sample was separately mixed with the quality control (QC) sample. A QC sample was run after every 5-15 analytical samples to test the stability of the whole detection process.

Chromatographic conditions were as follows: column: ACQUITY UPLC HSS T3 (100 mm × 2.1 mm i.d., 1.8 μm; Waters, Milford, MA, USA); mobile phase A: 95% water : 5% acetonitrile (containing 0.1% formic acid), mobile phase B: 47.5% acetonitrile : 47.5% isopropanol : 5% water (contains 0.1% formic acid); flow rate: 0.40 mL/min; injection volume: 2 μL; column temperature: 40°C. The gradient elution condition was: 95% A and 5% B, 0.1 min; 75% A and 25% B, 2 min; 100% B, 9 min; 100% B, 13 min; 100% A, 13.1 min; 100%, 16 min.

Mass spectrometry conditions were as follows: Samples were electrosprayed/ionized, and MS signals were collected in the negative ion scanning mode. Scan type (m/z): 70–1,050; Sheath gas flow rate (arb): 40; Aux gas flow rate (arb): 10; Heater temp (°C): 400; Capillary temp (°C): 320; Spray voltage (–) (V): −2,800; S-Lens RF Level: 50; Normalized collision energy (eV): 20, 40, 60; Resolution (Full MS): 70000; Resolution (MS2): 17500.

### Statistical analysis

All samples were tested in triplicates. Significant differences between data were identified by ANOVA Tukey’s test. Origin 9.8.5 was used for plotting the bar charts, stacked histograms, etc. Heatmaps were plotted by R Version 3.6.3 to analyze the changes. Adobe Illustrator 2020 was used to map the metabolic pathways.

## Results and discussion

### Analysis of the physicochemical indicators of RJ and *Lactobacillus plantarum fermented juice*

**FIGURE 1:**
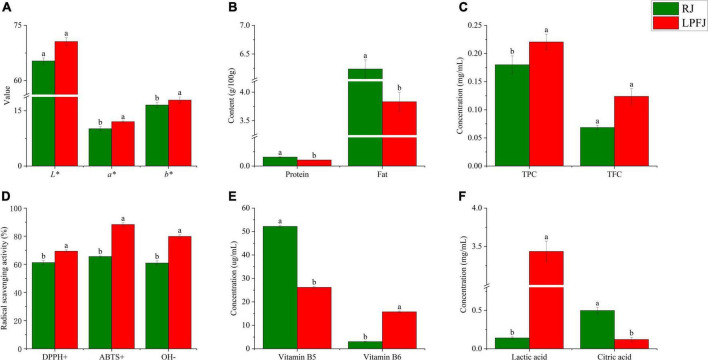
Analysis of the physicochemical indicators of RJ and *Lactobacillus plantarum* fermented juice (LPFJ). Post-LAB fermentation change in panel **(A)** color properties of chickpea milk L* (brightness), a* (red-green), and b* (yellow and blue), **(B)** protein and fat contents, and functional indicators including **(C)** TPC and TFC, **(D)** radical scavenging activity, **(E)** vitamin B5 and vitamin B6 contents, and **(F)** lactic and citric acids contents. Values marked with different superscript letters indicate statistically significant differences (*p* < 0.05). RJ, raw juice; LPFJ, *L. plantarum* fermented juice; TPC, total phenol content; TFC, total fat content.

Food color is a critical indicator of food quality (34). The mean L*, a*, and b* values of different samples are shown in Figure 1A. Compared with RJ (raw juice), the change in b* and a* values indicated a significant color change of LPFJ (*L. plantarum* fermented juice), however, the L* value did not change much (*p* < 0.05). The LAB fermentation changed the color of chickpea milk to more red and yellow colors.

Also, after fermentation, both total protein and total fat contents showed a downward trend (Figure 1B, *P* < 0.05). The decrease in total protein content may be because LAB promoted protein degradation during fermentation to meet the bacterial needs for nitrogen-containing substances (35). The lactic acid bacteria-degraded proteins are easily absorbed by the body; the formed ACE (angiotensin-converting enzyme) inhibiting peptides have anti-cardiovascular disease and blood pressure-lowering activities (36). Regarding the total fat content, its decrease may be due to the content of linoleic and linolenic acid decreased (37). The linolenic acid in soymilk positively correlates with the content of hexanal, which produces beany flavor (38). Therefore, LAB fermentation should reduce the beany flavor of chickpea milk. Hence, a decrease in total protein and total fat contents improved human digestion/absorption and the flavor of LPFJ.

The post-fermentation change in microorganism number is significantly increased (*p* < 0.05). The results indicated that LAB had good growth in chickpea milk and rapidly accumulated after 24 h of fermentation, reaching 8.87 log_10_CFU/mL. Consistently, past research has also demonstrated that LABs grew better in soymilk with microbiological counts ranging from 5.7 to 10.4 log_10_CFU/mL (39). Overall, chickpea milk is a suitable fermentation substrate with enough nutrients for the good growth of LAB.

As shown in Figures 1C,D, LAB fermentation significantly enhanced the TPC and DPPH+, ABTS+, and OH– scavenging capacity of LPFJ (*p* < 0.05), indicating a positive effect. However, TFC did not change much (*p* ≥ 0.05). Increased phenolic content can be attributed to the production of β-galactosidase during LAB fermentation, which catalyzes the release of phenolic compounds from bound sugars increasing antioxidant activity (40). Also, there could be other microbial transformations and depolymerization of compounds (41). Next, a small increase in TFC may be because LAB promote the release of flavonoids by producing enzymes and acids. *Lactobacillus plantarum* has been shown to increase TFC during fermentation (42).

Hydrolysis of glucosides through enzymatic processes using β-glucosidase to increase their bioavailability in soy-based products, and probiotic micro-organisms have been found to possess β-glucosidase (43, 44). The results showed a significant increase in β-glucosidase activity measured after fermentation (*p* < 0.05), reaching 28.13 mU/mL. Remarkably, the higher β-glucosidase activity in fermented soymilk indicates that the strain converts glycosidic soy isoflavones to glycosidic soy isoflavones faster, and the antioxidant capacity is enhanced (27, 44, 45). Subsequently, we utilized three different methods to assess the antioxidant properties of LPFJ (Figure 1D). The enhancement of antioxidant capacity indicates the accumulation of antioxidant compounds, such as phenolic compounds, flavonoids, and superoxide dismutase (SOD) (46). The lactic acid bacteria-fermented fruit and vegetable juices also show an increase in free radical scavenging and antioxidant activity (47, 48). Compared with RJ, LPFJ showed a significant increase in antioxidant activity, which may promote its health benefits in daily use (49).

Besides having abundant proteins, chickpeas are also rich in vitamins and minerals, especially vitamins B5 and B6 (50). Here, we found that the content of vitamin B5 decreased and the content of vitamin B6 increased significantly in LPFJ after LAB fermentation (*p* < 0.05; Figure 1E). Consistently, past research has also indicated that LAB fermentation can increase the contents of vitamin B6 in soymilk (51). The lactic acid bacterias are considered the biological factories for vitamin B production, and utilization (52). During LAB fermentation, lactic acid, a major organic acid in fermented beverages, increases (53). Meanwhile, the content of citric acid, which is an intermediate metabolite of the tricarboxylic acid cycle (TCA cycle), shows a dynamic change. The TCA cycle is the pivot for the liaison and transformation of key substances, such as sugar, lipid, protein, and even nucleic acids. Therefore, we measured the change in contents of lactic and citric acids (Figure 1F). Lactic acid production is not limited to sugars and citric acid as it may also originate from the conversion of amino acids (54). Here too, the lactic acid content increased and the citric acid content decreased in LPFJ.

### Change in aroma profiles of RJ and *Lactobacillus plantarum* fermented juice

#### Types, content, and relevance of aroma substances in RJ and *Lactobacillus plantarum* fermented juice

**FIGURE 2:**
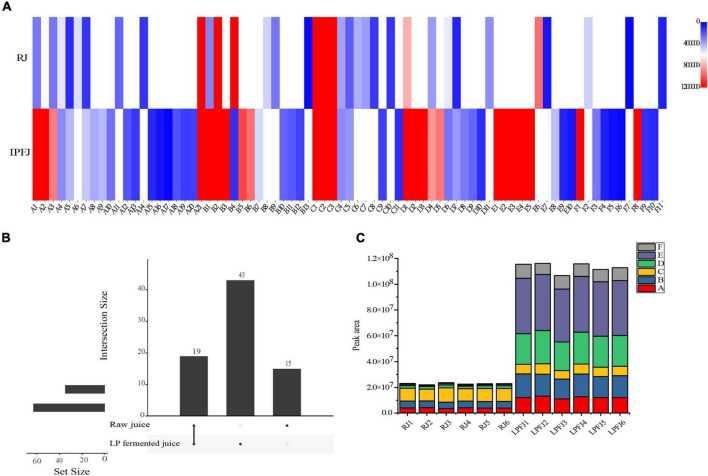
Change in aroma profiles of RJ and *Lactobacillus plantarum* fermented juice (LPFJ). **(A)** Content of aroma substances: the degree of redness and blueness indicate the higher and lower content, respectively. **(B)** Quantity of aroma substances: the value represents the type and a larger value indicates the sample richness of that substance. **(C)** A stacked histogram of volatile contents: A-alcohol; B-aldehyde; C-ester; D-acid; E-ketone; and F-others. RJ, raw juice; LPFJ, *L. plantarum* fermented juice.

The post-fermentation change in aroma compounds was determined by HS-SPME–GC/MS; in total, 77 aroma compounds were measured, containing 21 alcohols, 13 aldehydes, 11 esters, 10 ketones, 11 acids, and 11 others (Figure 2A and Supplementary Table 2). Aroma compounds followed a kinetic trend during the fermentation. There were 19 common substances, while 43 new substances were produced in LPFJ, and 15 substances were present only in RJ (Figure 2B). This indicated that LAB fermentation transformed substances in RJ producing a series of new flavor substances in LPFJ (55).

Figure 2A (A1–A21) shows the significant change in alcohols after LAB fermentation (*p* < 0.05); the enzyme activity in LPFJ increased by nearly 50%, while the half-life decreased (56). This significantly improved the total alcohol content in LPFJ. The lactic acid bacteria fermentation produces more alcohol substances. Hexanol (A2) is a typical feature of legume flavor compounds, with a fruity aromatic aroma; 1-nonanol (A14) has a slightly pleasant aroma of rose and orange. The production and accumulation of these substances improved the flavor of fermented soymilk (57). Also, 1-octen-3-ol (A1) is a common volatile organic compound in fermented soy products, which is generated by unsaturated fatty acid metabolism (58).

The main aldehyde and ketone substances in soymilk were acetone, 2-butanone, 3-methyl-butyraldehyde, and hexanal. The flavor substances and soy protein can bind hydrophobically, and their binding constants increase with the number of carbon chains (59). This complicates the elimination of beany flavor during the fermentation. Our results showed that the content of hexanal (B4) decreased significantly after LAB fermentation (*p* < 0.05; Figure 2A and Supplementary Table 2). Hexanal could have been converted to hexanoic acid eliminating the beany flavor and improving the flavor of chickpea milk (60). Also, there were high levels of benzaldehyde (B2), 3-hydroxy-butanal (B13), acetone (E6), and others in RJ. Meanwhile, levels of certain substances increased in LPFJ, most are ketones. These can be attributed to amino acids degradation by microbial metabolism (61, 62). Furthermore, the increased content of octaldehyde (B5) added a certain fruity flavor to LPFJ. Also, the reduction in acetone with special odors (spicy and sweet) greatly improved the flavor of the beverage increasing consumer demand for taste.

Typically, some acids undergo esterification to form esters, which provide a fruity or floral aroma and contribute to the mild and pleasant flavors of fermented foods (63). As shown in Figure 2C, the total content of esters did not change much, however, a general trend showed the production of new substances and the decrease/disappearance of old ones (Figure 2A). Notably, the flavor of LPFJ would have improved due to the peach aroma of 2-phenylethyl acetate (C11) and the pineapple aroma of 2-phenylethyl caproate (C9) produced after fermentation. And, LAB fermentation also increased the acid content in chickpea milk, especially acetic and nonanoic acids.

Next, we performed correlation analyses between the flavor substances (Supplementary Figure 1). We found significant positive linear correlations between alcohols and aldehydes, and aldehydes and acids (*p* < 0.05). Additionally, both alcohols and acids had a significant negative correlation with esters (*p* < 0.05). Typically, alcohols and acids undergo esterification and therefore have a negative correlation (63). Also, aldehydes and ketones showed a negative correlation with esters. Furthermore, LAB fermentation led to obvious differences in the types of flavor and specific substances between LPFJ and RJ. The changes in the relative content of each component directly affect the flavor of chickpea milk, such as the decrease in beany flavor after LAB fermentation.

#### Key aroma compounds in RJ and *Lactobacillus plantarum* fermented juice

**FIGURE 3:**
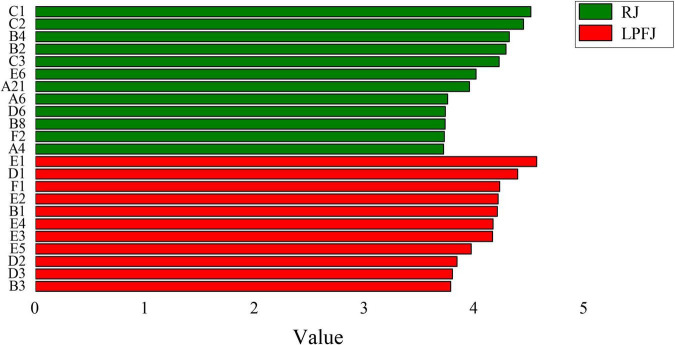
Linear discrimination analysis and the Kruskal-walls test to find significantly different aroma species (LDA score). A volatile compound with an LDA score > 3.7 was considered a significantly different species and a biomarker of soymilk fermentation. RJ, raw juice; LPFJ, *L. plantarum* fermented juice.

The key aroma compounds are the main components of fermented chickpea milk, which determine the aroma characteristics and form the unique flavor of fermented chickpea milk. We found clear enrichment of a few aroma substances to different levels in LPFJ. Most aldehydes were abundant in RJ (Figure 3). The key aroma substances in RJ were mainly hexanal (B4), benzaldehyde (B2), 1,4-butanediol (A6), etc. Some of these have a grassy, bitter almond flavor. The main compounds in LPFJ were ketones [2-heptanone (E1), 2-nonanone (E3), etc.] and acids [acetic acid (D1), 3-methyl-butanoic acid (D2), hexanoic acid (D3)]. Also, a flavor substance with a faint “smelly foot smell” was produced in LPFJ. Based on existing literature, this substance could be isovaleric acid, which is a common characteristic flavor component in fermented soybean products (64). Besides, LPFJ had some special substances with relatively high content, such as (E)-2-heptenal (B1), (E)-2-octenal (B3), etc. (E)-2-heptenal has a little fatty aroma; 2-heptanone and 2-nonanone have a fruity and creamy aroma. Acetoin (E2) improves the overall sensory acceptability of fermented soymilk (65). Due to carbonyl compounds could be converted by lactic acid bacterial metabolism into further well-known odor substances, like ketones, esters, or organic acids (66). So, post-fermentation, the good aroma substance increased in LPFJ, while the beany flavor weakened, improving the total flavor of the beverage.

### Metabolite analysis: Untargeted metabolomics

#### Types, classification, and differential metabolites in RJ and *Lactobacillus plantarum* fermented juice

Based on the untargeted metabolomic approach, the comprehensive changes in metabolites were analyzed to identify differential metabolites between RJ and LPFJ. In total, 218 metabolites were detected by UHPLC (Supplementary Figure 2). The levels of substances changed significantly during fermentation (*p* < 0.05), indicating a mutual transformation of substances. All metabolites detected in RJ and LPFJ were matched with the human metabolome database (HMDB). The changed metabolites belonged to nine primary classifications, 32 secondary classifications, and 51 tertiary classifications, and some were not identified. The dynamic changes in metabolite contents between RJ and LPFJ are shown in Supplementary Figure 3. At the level of primary classification, the metabolites were organoheterocyclic compounds (oxygenates 11.9%), lipids and lipid-like molecules (32.6%), organic acids and derivatives (17.9%), and so on. The majority of organic acids and derivatives were amino acids, peptides, and analogs (71.8%); organic oxygenates were composed of carbohydrates and carbohydrate conjugates (92.3%). Also, a significant increase in the content of phenylpropanoids and polyketides, lipids, and lipid-like molecules, and the total content of metabolites was observed in LPFJ (*p* < 0.05), indicating the role of LAB fermentation.

**FIGURE 4:**
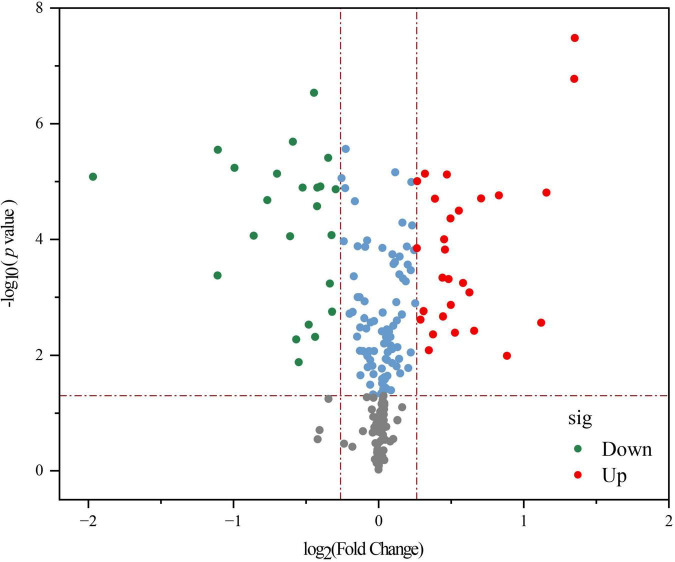
A volcano plot showing differential non-volatile metabolites between RJ and *Lactobacillus plantarum* fermented juice (LPFJ). Red and green indicate up-and down-regulated metabolites.

A volcano diagram shows the overall distribution of significantly different metabolites (*p* < 0.05) in Figure 4. Based on threshold criteria *p* < 0.05 and FC (fold change) ≥ 1.2, we found 28 up-regulated and 23 down-regulated metabolites in LPFJ after fermentation. The up-regulated metabolites were pyridoxine (vitamin B6), indole acetaldehyde, 5-L-glutamyl-L-alanine, 5-hydroxy-indole-acetaldehyde, and others. Notably, the increase in vitamin B6 is consistent with the results in Figure 1E. The down-regulated substances were xanthosine, vanilloside, D-glucarate, and others. Based on the Kyoto Encyclopedia of Genes and Genomes (KEGG) pathway descriptions, indole acetaldehyde and 5-hydroxy-indole-acetaldehyde participate in amino acid metabolism; 5-L-glutamyl-L-alanine is involved in other amino acid metabolism; Xanthosine is involved in nucleotide metabolism, biosynthesis of other secondary metabolites and membrane metabolism; D-Glucarate is involved in carbohydrate metabolism.

#### Key metabolites in RJ and *Lactobacillus plantarum* fermented juice

**FIGURE 5:**
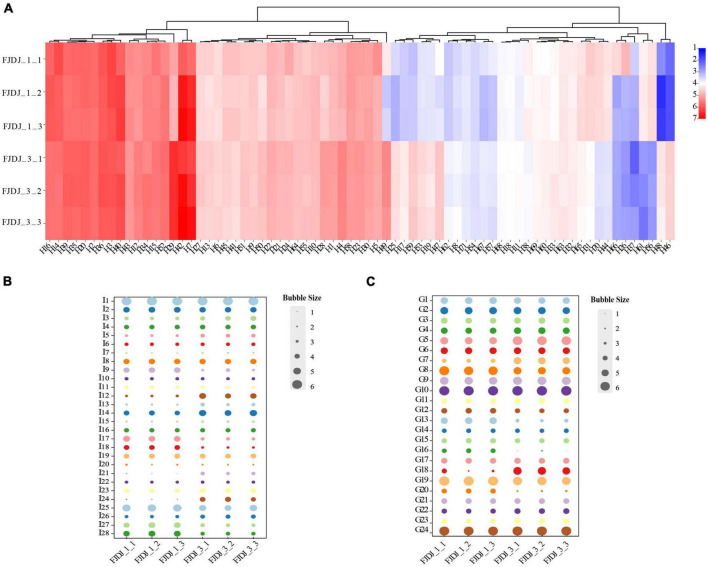
Key metabolites in RJ and *Lactobacillus plantarum* fermented juice (LPFJ). Content changes of **(A)** lipids and lipid-like molecules, **(B)** amino acids, peptides, and analogs, and **(C)** carbohydrates and carbohydrate conjugates. FJDJ1-1, FJDJ1-2, FJDJ1-2 belong to RJ; FJDJ3-1, FJDJ3-2, and FJDJ3-2 belong to LPFJ.

Legumes are rich in proteins and carbohydrates and are an excellent source of unsaturated fatty acids (50). The change in these substances indicated that LAB fermentation effectively worked in soymilk, improving its digestion, absorption, nutritional, and functional qualities (67). Carbohydrates in fermented beverages can promote colon health (68). Therefore, we next analyze the change in these metabolites (Figure 5).

**FIGURE 6:**
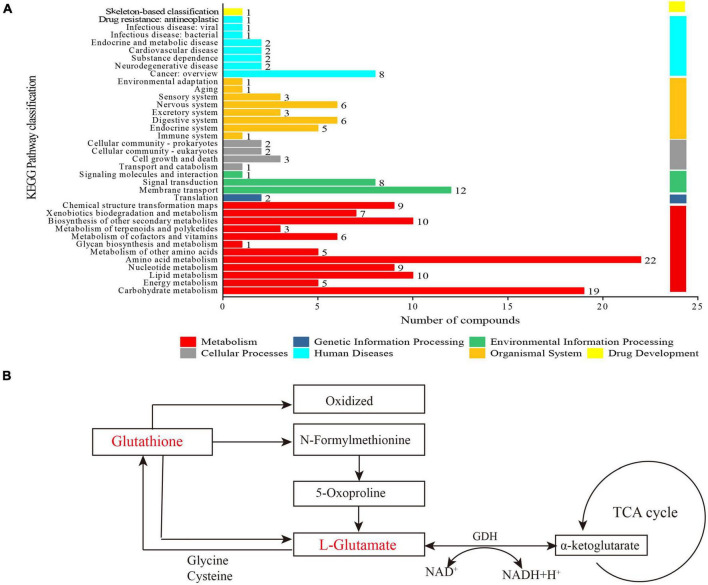
Metabolic pathways analysis. **(A)** Kyoto Encyclopedia of Genes and Genomes (KEGG) metabolite statistics; **(B)** schematic diagram of the metabolic pathway for the selected metabolites.

The content of fatty acids changed in LPFJ during LAB fermentation (Figure 6A). There were 71 key metabolites, of which, 50 increased including 13 with a significant increase (*p* < 0.05), such as 9,10,13-trihydroxystearic acid (H25), 2-hydroxyhexadecanoic acid (H47), 7-methylinosine (H62), and so on. Studies have shown that the acid compounds generated during fermentation enhance the taste, flavor, and texture of the fermented product (68). In total, 21 metabolite decreased after fermentation, including 13 with a significant decrease (*p* < 0.05). Among them, PE (16:0/0:0) (H45) is an important phospholipid that constitutes biological membranes, mainly in the brain, nerves, microorganisms, and soybeans. It plays an important role in signal transduction and maintenance of life functions. LysoPC [18:2 (9Z, 12Z)] (H14) functions in lipid signaling by acting on the lysophospholipid receptor (LPL-R). There were also some substances involved in oleic and linoleic acid metabolism. Oleic and stearic acids are regarded as health-promoting substances for their preventive effects against high blood pressure and cardiovascular diseases (33).

The increase in peptides and amino acids is a result of soy protein hydrolysis by extracellular proteases of LAB. Similarly, we found an increase in the contents of amino acids, peptides, and analogs, including 9 with a significant increase (*p* < 0.05; Figure 5B). In general, LAB-fermented products are rich in beneficial peptides and amino acids (69). And study showed that *L. plantarum* has a high potential for peptide production (9). Notably, the contents of L-glutamine (I12), *N*-formylmethionine (I21), 5-L-glutamyl-L-alanine (I24), allysine (I26), and others almost doubled in LPFJ. Based on the KEGG database, these metabolites are involved in alanine, aspartate, and glutamate metabolism; the biosynthesis of arginine; protein digestion and absorption; metabolism of cysteine and methionine; metabolism of glutathione; degradation of lysine, etc. Additionally, there is the possibility that some peptides may provide a bitter taste to fermented products.

Change in carbohydrate content was observed throughout the fermentation process (Figure 5C). Carbohydrate metabolism produces intermediate products that are components of the bacterial cell structure, as well as provide energy for bacterial growth and survival. *L. plantarum* bacterium harbors multiple sugar metabolic pathways that can metabolize various sugars (70, 71). During the LAB fermentation, some carbohydrates such as ribonolactone (G5), ribitol (G7), and dulcitol (G22) increased significantly, while some such as jasmolone glucoside (G13), galactinol (G19), D-glucarate (G20) decreased (*p* < 0.05). Notably, galactinol is a common component of the cottonseed glycoconjugate family and participates in the biosynthesis of oligosaccharides. The beany flavor and high levels of nondigestible oligosaccharides are the key issues limiting the consumer acceptance of soy products. A decrease in galactinol can reduce the biosynthesis of oligosaccharides enhancing the digestibility of LPFJ.

#### Metabolic pathways analysis

In Figure 6A, partial metabolites were engaged in 37 metabolic pathways belonging to seven categories, such as organismal systems, human diseases, cellular processes, metabolism, and so on. These metabolic pathway categories were searched against the KEGG database. Among them, carbohydrate metabolism (19 metabolites), lipid metabolism (10 metabolites), amino acid metabolism (22 metabolites), biosynthesis of other secondary metabolites (10 metabolites), and membrane transport (10 metabolites) were the main metabolic pathways during the fermentation. Up to 22 kinds of metabolites were associated with amino acid metabolism. The lactic acid bacteria degrade proteins and peptides to meet the needs of bacterial growth (35). Additionally, certain peptides work on target sites for specific functions, such as antioxidant and anti-hypertension activities (72, 73). In summary, KEGG metabolite analysis revealed the diversity of metabolic pathways in LPFJ during LAB fermentation.

The levels of glutathione and L-glutamate decreased significantly after fermentation (*p* < 0.05; Figure 6B). Glutathione is broken down into *N*-formylmethionine and L-glutamate and oxidized. Glutamate, cysteine, and glycine are synthesized into glutathione, which is part of almost every cell in body (Figure 6B). Subsequently, glutamate is transformed into α-ketoglutarate (α-KG) by glutamate dehydrogenase (GDH), which is a part of the TCA cycle (Figure 6B). Glutathione is the richest non-protein thiol compound in all organisms (74). It has critical functions with antioxidant properties such as preventing cellular damage caused by various oxidative stressors, detoxification of pathogens, and immune enhancer (74, 75). Moreover, glutamate participates in many important metabolic reactions in animals, plants, and microorganisms. Therefore, an increase in glutathione improves the antioxidant capacity and other functional properties of fermented chickpea milk.

## Conclusion

In this study, chickpea milk was fermented using LP-56. The lactic acid bacteria fermentation improved the physicochemical characteristics, antioxidant activity, and other functional properties of fermented chickpea milk. Also, the content of beany substance was reduced improving the flavor of the final product. The increase in the content of acid and other substances improved the overall taste of LPFJ. Notably, 51 differential metabolites were identified between RJ and LPFJ that participate in multiple metabolic pathways providing the specific sensory and functional characteristics to LPFJ. An increase in glutathione metabolism also played an extremely important role. Our study uncovered the effect of LAB fermentation on chickpea milk in terms of flavor and functional properties, which may help the development and commercialization of fermented chickpea milk. We should next assess the changes in micronutrients (such as peptides) and identify their effect on the functional properties of LPFJ.

## Data availability statement

The original contributions presented in this study are included in this article/Supplementary material, further inquiries can be directed to the corresponding author.

## Author contributions

PZ: conceptualization, methodology, investigation, and writing—original draft. WC: investigation and software. XZ: investigation. FT: supervision. CS: project administration and funding acquisition. All authors contributed to the article and approved the submitted version.

## Supplementary material

Supplementary Figure 1 Correlation between substances: red is positive correlation, blue is negative correlation; the size of the circle represents the degree of correlation (*p* < 0.05).

Supplementary Figure 2 Heatmap of hierarchical clustering analysis of differential abundant metabolites in RJ and LPFJ.

Supplementary Figure 3 Sankey plot of the changes of non-volatile metabolites in RJ and LPFJ based on HMDB super class.
