# Supplementary material for: Evaluating the effect of lactic acid bacteria fermentation on quality, aroma, and metabolites of chickpea milk
Source: Front Nutr. 2022 Dec 5;9:1069714. doi: 10.3389/fnut.2022.1069714 (PMC9760965; doi:10.3389/fnut.2022.1069714)
Supplement: Supplementary file 1 [file Table_1.DOCX]

**Supplementary Text S1.**

1. Determination of total protein—Spectrophotometric method

Sample digestion: weigh 5 g of sample (accurate to 0.001 g), transfer into a dry 100 mL nitrogen fixing bottle, add 0.1 g CuSO_4_, 1 g K_2_SO_4_ and 5 mL H_2_SO_4_, shake well and place a funnel at the mouth of the bottle, support the nitrogen fixing bottle obliquely at an angle of 45° on an asbestos net with small holes, heat slowly, wait until the contents are all charred and the foam stops completely, strengthen the fire, and keep the liquid in the bottle slightly boiling, until the liquid is blue-green clarified and transparent, then continue to heat for 30 min, put down the cooling, slowly add 20 mL of water, let cool and then transfer into a 50 mL volumetric flask, fix the volume, mix and set aside. According to the same method to do reagent blank test.

Sample solution preparation: remove 5 mL of sample or reagent blank digestion solution in a 50 mL volumetric flask, add 2 d p-Nitrophenol indicator solution (1 g/L), shake well and add NaOH solution (300 g/L) dropwise to neutralize to yellow, then add acetic acid solution (1 mol/L) dropwise to the solution colorless, dilute with water to the scale, mix well.

Sample determination: Draw 2 mL of sample solution and reagent blank solution, put them in 10 mL test tubes, add 4ml of sodium acetic acid-acetic acid buffer solution and 4 mL of color developer, add water to the scale, and mix well. Place in a water bath at 100 ℃ for 15 min. remove and cool to room temperature with water, transfer to a cuvette and measure the absorbance at 400 nm.

Standard curve plotting: aspirate different volumes of ammonia nitrogen standard solution (in terms of nitrogen, 1 g/L) (equivalent to 0, 5, 10, 20, 40, 60, 80, 100ug nitrogen) with the above steps, according to the standard absorbance value of each point to plot the standard curve.

$$X=\frac{（C-C_{0})\times V_{1}\times V_{3}}{m\times V_{2}\times V_{4}\times1000\times1000}\times100\times F$$

X—Protein content in the sample, g/100g

C—The content of nitrogen in the specimen determination solution, µg

C_0_—The content of nitrogen in the reagent blank assay solution, µg

V_1_—Volume of sample digestion solution fixation, ml

V_3_—Total volume of sample solution,ml

m—Mass of samplen, g

V_2_—Volume of digestion solution for preparing the specimen solution,ml

V_4_—Volume of sample solution for determination,ml

1000—Conversion factor

100—Conversion factor

F—Conversion factor of nitrogen to protein

1. Determination of total fat - acid hydrolysis method

Hydrolysis of the sample: weigh 10 g, accurate to 0.001 g, put it in a 50 mL test tube, add 10 mL HCl (2 mol/L), put the test tube in a water bath at 70-80 ℃, and stir once every 5-10 min with a glass rod until the sample is completely digested, about 40-50 min.

Extraction: remove the test tube, add 10 mL of ethanol, mix, cool the mixture into a 100 mL stoppered cylinder, rinse the test tube with 25 mL of petroleum ether in several times, and pour it into the cylinder together, after all the petroleum ether is poured into the cylinder, add the stopper and shake for 1 min, carefully open the stopper, release the gas, then stopper it, leave it for 12 min, carefully open the stopper, and rinse the stopper and the mouth of the cylinder attached with petroleum ether fat. Let it stand for 10-20 min, when the upper liquid is clear, aspirate the supernatant in the conical flask with constant weight, then add 5 mL of petroleum ether in the measuring cylinder with stopper, shake, let it stand, aspirate the upper layer and put it into the original conical flask.

Weighing: Place the conical flask in 105 ℃ oven to dry for about 1 h, cool in the desiccator for 30 min, repeat the above steps until constant weight.

$$X=\frac{m_{1}-m_{0}}{m_{2}}$$

X—Content of fat in the sample, g/100g

m_1_—Conical flask and fat content after constant weight, g

m_0_—Mass of the conical flask, g

m_2_—Mass of the sample, g

100—Conversion factor
