# Supplementary material for: Evaluating the effect of lactic acid bacteria fermentation on quality, aroma, and metabolites of chickpea milk
Source: Front Nutr. 2022 Dec 5;9:1069714. doi: 10.3389/fnut.2022.1069714 (PMC9760965; doi:10.3389/fnut.2022.1069714)
Supplement: Supplementary file 2 [file Table_2.DOCX]

**Supplementary Text S2.**

1. Determination of total phenol

Take 0.8 mL of supernatant in 25 mL tube, then add distilled water to 10 mL, add 1 mL of Folin Phenol Reagent, mix well, keep away from light for 5 min, add 2 mL of 12 % Na_2_CO_3_ solution, then fix the volume with distilled water, keep away from light for 90 min at 23 ℃, measure the absorbance value at 765 nm with UV spectrophotometer. The standard curve was plotted with the concentration of gallic acid standard solution as the horizontal coordinate and the absorbance value as the vertical coordinate. Gallic acid standard concentration 0, 0.001, 0.002, 0.003, 0.004, 0.005, 0.006 mg/mL.

1. Determination of total flavonoids

Take 1 mL of supernatant in a 10 mL tube, add 60 % ethanol to make up to 5 mL, add 0.3 mL of 5 % NaNO_2_ solution, shake well, stand for 6 min, add 0.5 mL of 10 % Al_2_(NO_3_)_3_ solution, react for 6 min, then add 4 mL of 4 % NaOH solution, finally use 60 % ethanol to fix the volume to the scale, shake well, stand for 12 min The absorbance value at 510 nm was measured by UV spectrophotometer. The standard curve was plotted with the concentration of rutin as the horizontal coordinate and the absorbance value as the vertical coordinate. Rutin standard concentration 0, 0.2, 0.4, 0.6, 0.8, 1.0mg/mL.
