# Supplementary material for: Evaluating the effect of lactic acid bacteria fermentation on quality, aroma, and metabolites of chickpea milk
Source: Front Nutr. 2022 Dec 5;9:1069714. doi: 10.3389/fnut.2022.1069714 (PMC9760965; doi:10.3389/fnut.2022.1069714)
Supplement: Supplementary file 3 [file Table_3.DOCX]

**Supplementary Text S3.**

1. Determination of B5

10 g of the soymilk sample was added in a 100 mL conical flask with stopper and added warm water to 30 mL. Followed by the addition of 0.2 g amylase (≥1.5 U/mg). Shaken well and put stopper. Enzymolysis and shaken for 120 - 240 min under the condition of 55 ± 5℃ water bath. Then, cooled down to room temperature, adjusted pH to 5.0 ± 0.1 with 0.1 mol/L HCl, added 5ml 0.5 mol/L ZnSO_4_ solution, shaken well. Finally, the liquid was transferred into a 50 mL volumeter flask, ultrapure water was added to make up the volume, centrifuged at 3000 r for 10 min. The supernatant was passed through 0.45 μm water phase filter membrane as the test solution.

1. Determination of B6

10 g of the soymilk sample was added in a 150 mL conical flask with stopper. Followed by the addition of 0.5 g amylase. Shaken well and put stopper. Place in a 50 - 60 °C incubator for about 30 min. Then, cooled down to room temperature, Adjusted pH to 1.7 ± 0.1 with 5 mol/L HCl, Place for 1 min. Adjusted pH to 4.5 ± 0.1 with 5 mol/L NaOH, ultrasonic oscillation for 10 min. Finally, the liquid was transferred into a 50 mL volumeter flask, make up the volume. Filter paper filtration, The filtrate was passed through 0.45 μm water phase filter membrane as the test solution.
