# Supplementary material for: Evaluating the effect of lactic acid bacteria fermentation on quality, aroma, and metabolites of chickpea milk
Source: Front Nutr. 2022 Dec 5;9:1069714. doi: 10.3389/fnut.2022.1069714 (PMC9760965; doi:10.3389/fnut.2022.1069714)
Supplement: Supplementary file 4 [file Table_4.DOCX]

**TABLE S2**

**Supplementary Table S2 Peak area of Key metabolites in RJ and LPFJ**

| Code | Metabolite | Retention time | RJ | LPFJ |
| --- | --- | --- | --- | --- |
|  | Lipids and lipid-like molecules |  |  |  |
| H1 | 3-hydroxy-3-methyl-Glutaric acid | 1.6 | 4.82±0.03 | 5.14±0.03 |
| H2 | Sandosaponin A | 4.89 | 5.72±0.06 | 5.73±0.01 |
| H3 | Pisumsaponin II | 6.39 | 6.13±0.09 | 5.97±0.04 |
| H4 | Dehydrosoyasaponin I | 5.91 | 4.93±0.07 | 5.45±0.01 |
| H5 | 2-Isopropylmalic acid | 2.38 | 5.05±0.23 | 5±0.03 |
| H6 | (3S,5R,6R,7E)-3,5,6-Trihydroxy-7-megastigmen-9-one | 2.4 | 4.41±0.04 | 4.51±0.04 |
| H7 | PE(15:0/22:2(13Z,16Z)) | 8.66 | 6.3±0.52 | 6.48±0.33 |
| H8 | 6-(2-Hydroxyethoxy)-6-oxohexanoic acid | 2.4 | 3.58±0.07 | 3.91±0.09 |
| H9 | Pisumic acid | 1.96 | 4.63±0.01 | 4.62±0.03 |
| H10 | (+/-)-Glycerol 1,2-diacetate | 1.71 | 4.81±0.07 | 4.79±0.03 |
| H11 | 1,11-Undecanedicarboxylic acid | 6.02 | 3.84±0.04 | 4.01±0.04 |
| H12 | PIP(16:0/22:5(4Z,7Z,10Z,13Z,16Z)) | 6.04 | 5.28±0.05 | 5.49±0.04 |
| H13 | 11-Oxohexadecanoic acid | 7.02 | 4.43±0.11 | 4.55±0.04 |
| H14 | LysoPC(18:2(9Z,12Z)) | 7.75 | 5.96±0.13 | 5.66±0.03 |
| H15 | LysoPC(18:1(9Z)) | 8.25 | 5.52±0.09 | 5.6±0.03 |
| H16 | 1-(sn-Glycero-3-phospho)-1D-myo-inositol | 0.64 | 5.91±0.09 | 5.68±0.01 |
| H17 | 8-[(Aminomethyl)sulfanyl]-6-sulfanyloctanoic acid | 1.56 | 3.39±0.02 | 4.23±0.05 |
| H18 | Pimelic acid | 2.53 | 3.95±0.02 | 4.01±0.02 |
| H19 | 20-hydroxy-PGE2 | 3.89 | 3.69±0.01 | 4.33±0.06 |
| H20 | Bersimoside I | 4.07 | 5.77±0.06 | 5.89±0.03 |
| H21 | Jujuboside C | 4.32 | 4.74±0.05 | 4.86±0.03 |
|  |  |  |  |  |
| Continued |  |  |  |  |
| Code | Metabolite | Retention time | RJ | LPFJ |
| H22 | Balanitesin | 4.32 | 4.84±0.06 | 4.95±0.03 |
| H23 | Corchorifatty acid F | 5.18 | 5.06±0.07 | 5.24±0.02 |
| H24 | 9,10,13-TriHOME | 5.46 | 4.74±0.23 | 4.85±0.03 |
| H25 | 9,10,13-Trihydroxystearic acid | 6 | 3.23±0.29 | 4.39±0.01 |
| H26 | Ganoderic acid Mi | 6.19 | 3.68±1.01 | 2.78±0.1 |
| H27 | Furanofukinin | 6.26 | 4.48±0.06 | 4.61±0.07 |
| H28 | 9,10-DiHOME | 7.07 | 4.62±0.13 | 5.17±0.03 |
| H29 | 5-Hexyltetrahydro-2-furanoctanoic acid | 7.68 | 4.93±0.11 | 6.46±0.01 |
| H30 | LysoPC(16:0) | 8.13 | 5.09±0.04 | 5.11±0.05 |
| H31 | 1-(11Z,14Z-eicosadienoyl)-glycero-3-phosphate | 8.22 | 4.54±0.08 | 4.03±0.03 |
| H32 | (S)-10,16-Dihydroxyhexadecanoic acid | 8.31 | 4.21±0.04 | 4.23±0.02 |
| H33 | Sagittariol | 8.92 | 4.05±0.03 | 4.26±0.05 |
| H34 | PC(18:2(9Z,12Z)/18:3(6Z,9Z,12Z)) | 9.09 | 5.45±0.05 | 5.57±0.14 |
| H35 | GPEtn(18:1/18:2) | 10.89 | 5.74±0.07 | 5.83±0.07 |
| H36 | GPEtn(18:2/18:2) | 10.27 | 5.94±0.05 | 6±0.07 |
| H37 | Stearaldehyde | 8.88 | 3.16±0.25 | 2.49±0.36 |
| H38 | 12-hydroxyheptadecanoic acid | 8.86 | 4.15±0.07 | 3.92±0.04 |
| H39 | NORETHINDRONE ACETATE | 8.83 | 5.7±0.06 | 5.86±0.05 |
| H40 | PI(16:0/18:2(9Z,12Z)) | 8.6 | 6.12±0.25 | 6.04±0.13 |
| H41 | 1-Palmitoylglycerophosphoinositol | 8.47 | 4.67±0.09 | 4.43±0.04 |
| H42 | PC(18:1(11Z)/18:2(9Z,12Z)) | 8.45 | 6.52±0.55 | 6.7±0.35 |
| H43 | 12-Hydroxy-8,10-octadecadienoic acid | 8.31 | 5.34±0.04 | 5.23±0.01 |
| H44 | 3-hydroxypentadecanoic acid | 8.31 | 4.31±0.04 | 3.67±0.02 |
|  |  |  |  |  |
| Continued |  |  |  |  |
| Code | Metabolite | Retention time | RJ | LPFJ |
| H45 | PE(16:0/0:0) | 8.13 | 4.67±0.09 | 4.52±0.07 |
| H46 | Armillaric acid | 7.68 | 2.04±0.17 | 4.55±0.01 |
| H47 | 2-hydroxyhexadecanoic acid | 7.42 | 3.76±0.21 | 4.66±0.01 |
| H48 | 13-Hydroxy-9-methoxy-10-oxo-11-octadecenoic acid | 7.27 | 1.96±0.6 | 4.26±0.08 |
| H49 | 9(S)-HODE | 7.27 | 3.72±0.47 | 5.35±0.07 |
| H50 | (9S,10S)-9,10-dihydroxyoctadecanoate | 7.25 | 4.48±0.15 | 4.76±0.05 |
| H51 | 12-Oxo-2,3-dinor-10,15-phytodienoic acid | 6.31 | 4.57±0.06 | 4.69±0.03 |
| H52 | Prenyl apiosyl-(1->6)-glucoside | 4.36 | 5.55±0.13 | 5.69±0.02 |
| H53 | 12-Oxo-20-trihydroxy-leukotriene B4 | 3.84 | 3.69±0.02 | 4.44±0.04 |
| H54 | Suberic acid | 3.21 | 3.54±0.01 | 3.63±0.03 |
| H55 | Alpha-Ionol O-[arabinosyl-(1->6)-glucoside] | 3.19 | 4.69±0.07 | 4.73±0.06 |
| H56 | (1S,2S,4R,8S)-p-Menthane-1,2,8,9-tetrol 2-glucoside | 2.94 | 4.4±0.05 | 2.8±0.07 |
| H57 | 3-Hydroxy-2-methylglutarate | 2.8 | 3.47±0.28 | 3.66±0.09 |
| H58 | Eremopetasidione | 2.69 | 5.17±0.02 | 5.2±0.02 |
| H59 | (1xi,2xi)-1-(4-Hydroxyphenyl)-1,2,3-propanetriol 3-O-beta-D-Glucopyranoside | 2.51 | 3.34±0.06 | 4.63±0.04 |
| H60 | 3-Methyl-3-butenyl apiosyl-(1->6)-glucoside | 2.47 | 4.08±0.08 | 4.19±0.01 |
| H61 | Oleoside 11-methyl ester | 2.2 | 4.22±0.04 | 2.59±0.21 |
| H62 | 7-Methylinosine | 2.09 | 3.37±0.03 | 3.83±0.07 |
| H63 | Gibberellin A1 glucosyl ester | 2 | 4.29±0.03 | 4.29±0.06 |
| H64 | Ethyl 4-(methylthio)butyrate | 1.71 | 4.71±0.02 | 4.75±0.02 |
| H65 | 3-Dehydronobilin | 1.71 | 4.4±0.06 | 4.11±0.04 |
| H66 | 4-Hydroxyproline galactoside | 1.71 | 3.49±0.85 | 2.96±0.01 |
|  |  |  |  |  |
| Continued |  |  |  |  |
| Code | Metabolite | Retention time | RJ | LPFJ |
| H67 | Adipic acid | 1.65 | 3.34±0.29 | 3.74±0.05 |
| H66 | 4-Hydroxyproline galactoside | 1.71 | 3.49±0.85 | 2.96±0.01 |
| H67 | Adipic acid | 1.65 | 3.34±0.29 | 3.74±0.05 |
| H68 | Propionylcarnitine | 1.52 | 3.91±0.01 | 3.98±0.02 |
| H69 | Melibiitol | 1.45 | 4.08±0.04 | 4.11±0.02 |
| H70 | 3-Chloro-2-methyldienelactone | 1.36 | 4.45±0.03 | 3.62±0.04 |
| H71 | Methyl jasmonate | 4.36 | 3.71±0.03 | 3.75±0.03 |
|  |  |  |  |  |
|  | Amino acids, peptides, and analogues |  |  |  |
| I1 | Gamma-Glutamylphenylalanine | 2.29 | 6.84±0.04 | 6.79±0.02 |
| I2 | Gamma-Glutamyltryptophan | 2.53 | 5.3±0.05 | 5.17±0.03 |
| I3 | Tyrosyl-Histidine | 3.21 | 4.13±0.03 | 4.74±0.24 |
| I4 | Arginyl-Asparagine | 3.66 | 4.61±0.03 | 4.69±0.03 |
| I5 | N-Acetyl-L-glutamic acid | 1.47 | 3.69±0.02 | 4.26±0.01 |
| I6 | Asparaginyl-Proline | 1.6 | 4.06±0.02 | 4.11±0.02 |
| I7 | Pantetheine | 2.13 | 2.97±0.05 | 3.1±0.02 |
| I8 | N-Phenylacetylaspartic acid | 2.65 | 5.11±0.04 | 5.08±0.04 |
| I9 | Tyrosyl-Aspartate | 1.71 | 5.14±0.09 | 3.81±0.35 |
| I10 | Pyrroline hydroxycarboxylic acid | 1.52 | 3.99±0.02 | 3.91±0.05 |
| I11 | N-Methylglutamic acid | 0.72 | 4.07±0.02 | 3.97±0.09 |
| I12 | L-Glutamine | 0.64 | 3.65±0.07 | 5.18±0.2 |
| I13 | N-acetylaspartate | 1.12 | 3.32±0.03 | 4.01±0.2 |
| I14 | L-4-Hydroxyglutamate semialdehyde | 1.43 | 4.84±0.11 | 5.51±0.15 |
|  |  |  |  |  |
| Continued |  |  |  |  |
| Code | Metabolite | Retention time | RJ | LPFJ |
| I15 | 3-Methylcrotonylglycine | 2.15 | 3.21±0 | 3.45±0.03 |
| I16 | Acetyl-DL-Leucine | 2.76 | 4.54±0.01 | 4.51±0.02 |
| I17 | N-Lauroylglycine | 7.13 | 5.32±0.09 | 3.97±0.06 |
| I18 | Myristoylglycine | 7.9 | 4.79±0.02 | 3.52±0.02 |
| I19 | Lysyl-Asparagine | 2.29 | 4.97±0.05 | 4.92±0.02 |
| I20 | Hydroxyprolyl-Valine | 2.02 | 3.14±0.16 | 3.07±0.04 |
| I21 | N-Formylmethionine | 2 | 2.84±0.17 | 4.12±0.12 |
| I22 | Creatine | 1.82 | 3.84±0.01 | 3.9±0.04 |
| I23 | N-lactoyl-Valine | 1.71 | 4.33±0.05 | 4.91±0.05 |
| I24 | 5-L-Glutamyl-L-alanine | 1.27 | 2.77±0.09 | 4.82±0.19 |
| I25 | Phytosulfokine b | 0.76 | 6.12±0.05 | 5.52±0.18 |
| I26 | Allysine | 1.67 | 4±0.05 | 4.43±0.04 |
| I27 | Glutathione | 1.21 | 5.24±0.2 | 4.2±0.14 |
| I28 | L-Glutamate | 0.64 | 5.33±0.11 | 4.26±0.04 |
|  |  |  |  |  |
|  | Carbohydrates and carbohydrate conjugates |  |  |  |
| G1 | Taxiphyllin | 2 | 5.15±0.04 | 4.96±0.03 |
| G2 | 3-Deoxy-D-glycero-D-galacto-2-nonulosonic acid | 0.72 | 5.33±0.04 | 5.22±0.1 |
| G3 | L-Ribulose | 0.74 | 4.64±0.07 | 4.78±0.06 |
| G4 | D-1-Deoxy-erythro-hexo-2,3-diulose | 0.74 | 4.77±0.06 | 4.98±0.08 |
| G5 | Ribonolactone | 1.21 | 5.3±0.06 | 6.2±0.01 |
| G6 | L-Galacto-2-heptulose | 0.74 | 5.04±0.02 | 5.04±0.03 |
| G7 | Ribitol | 0.68 | 3.71±0.06 | 5.23±0.12 |
|  |  |  |  |  |
| Continued |  |  |  |  |
| Code | Metabolite | Retention time | RJ | LPFJ |
| G8 | Gluconic acid | 0.66 | 6.19±0.19 | 5.39±0.01 |
| G10 | Arabinofuranobiose | 0.68 | 6.51±0 | 6.63±0.01 |
| G11 | L-Fucose | 1.03 | 4.39±0.07 | 4.54±0.05 |
| G12 | 1,2,10-Trihydroxydihydro-trans-linalyl oxide 7-O-beta-D-glucopyranoside | 2.8 | 4.53±0.08 | 4.1±0.04 |
| G13 | Jasmolone glucoside | 2.31 | 5.05±0.08 | 3.31±0.17 |
| G14 | Acetaminophen glucuronide | 1.82 | 3.86±0.06 | 3.8±0.06 |
| G15 | 3-Fucosyllactose | 1.8 | 4.22±0.11 | 4.11±0.08 |
| G16 | Vanilloside | 1.75 | 3.92±0.08 | 1.82±0.33 |
| G17 | B-D-Xylopyranosyl-(1->4)-a-L-rhamnopyranosyl-(1->2)-L-arabinose | 1.62 | 4.55±0.02 | 4.62±0.03 |
| G18 | Beta-D-Xylopyranosyl-(1->5)-alpha-L-arabinofuranosyl-(1->5)-L-arabinose | 1.25 | 2.97±0.94 | 5.49±0.15 |
| G19 | Galactinol | 0.74 | 6.49±0.04 | 5.94±0.15 |
| G20 | D-Glucarate | 0.66 | 4.14±0.05 | 2.44±0.12 |
| G21 | D-Glucose | 0.62 | 4.45±0.01 | 4.48±0.03 |
| G22 | Dulcitol | 0.64 | 4.14±0.01 | 4.48±0.02 |
| G23 | Glucosamine | 1.43 | 4.14±0.1 | 4.52±0.01 |
| G24 | Maltotriose | 0.74 | 6.38±0.05 | 6.46±0.01 |
