# Supplementary material for: Evaluating the effect of lactic acid bacteria fermentation on quality, aroma, and metabolites of chickpea milk
Source: Front Nutr. 2022 Dec 5;9:1069714. doi: 10.3389/fnut.2022.1069714 (PMC9760965; doi:10.3389/fnut.2022.1069714)
Supplement: Supplementary file 5 [file Table_5.DOCX]

**TABLE S1**

**Supplementary Table S1 Peak area of volatile compounds in RJ and LPFJ**

| Code | RT/min | Compounds name by classes | Formula | CAS | RI | Peak area of volatile compounds | |
| --- | --- | --- | --- | --- | --- | --- | --- |
|  |  |  |  |  |  | RJ | LPFJ |
|  |  | Alcohols |  |  |  |  |  |
| A1 | 28.051 | 1-Octen-3-ol | C8 H16 O | 3391-86-4 | 1438 | 281234±39618 | 1661498±40901 |
| A2 | 21.769 | 1-Hexanol | C6 H14 O | 111-27-3 | 1362 | ND | 1655337±120641 |
| A3 | 15.76 | 1-Pentanol | C5 H12 O | 71-41-0 | 1275 | 280562±23253 | 906929±114819 |
| A4 | 56.564 | Phenylethyl Alcohol | C8 H10 O | 60-12-8 | 1930 | 541998±15883 | 323822±40149 |
| A5 | 57.921 | Maltol | C6 H6 O3 | 118-71-8 | 1962 | 101853±13046 | 431707±49632 |
| A6 | 57.216 | 1,4-Butanediol | C4 H10 O2 | 110-63-4 | 1941 | 520990±83831 | ND |
| A7 | 30.766 | 1-Hexanol, 2-ethyl- | C8 H18 O | 104-76-7 | 1501 | 124872±12218 | 466002±33357 |
| A8 | 55.458 | Benzyl alcohol | C7 H8 O | 100-51-6 | 1880 | ND | 397894±34085 |
| A9 | 28.231 | 1-Heptanol | C7 H16 O | 111-70-6 | 1442 | ND | 434698±31736 |
| A10 | 15.729 | 3-Buten-1-ol, 3-methyl- | C5 H10 O | 763-32-6 | 1272 | ND | 267594±29163 |
| A11 | 63.169 | 1,2,3-Butanetriol | C4 H10 O3 | 4435-50-1 | 2274 | 238372±13975 | ND |
| A12 | 35.429 | Linalool | C10 H18 O | 78-70-6 | 1549 | ND | 199351±7873 |
| A13 | 40.962 | 2-Octen-1-ol, (Z)- | C8 H16 O | 26001-58-1 | 1610 | ND | 129045±9953 |
| A14 | 62.015 | 1,5-Anhydro-d-mannitol | C6 H12 O5 | 492-93-3 | 2150 | 119920±6722 | ND |
| A15 | 44.902 | 1-Nonanol | C9 H20 O | 143-08-8 | 1660 | ND | 90926±7282 |
| A16 | 21.251 | 2-Heptanol, 5-methyl- | C8 H18 O | 54630-50-1 | 1354 | ND | 46976±5855 |
| A17 | 21 | Cyclohexanol, 2,4-dimethyl- | C8 H16 O | 69542-91-2 | 1352 | ND | 12878±1696 |
| A18 | 41.712 | Isomaltol | C6 H6 O3 | 3420-59-5 | 1615 | ND | 191520±10576 |
| A19 | 11.91 | Bicyclo[2.1.1]hexan-2-ol, 2-ethenyl- | C8 H12 O | 1000221-37-2 |  | ND | 143429±11606 |
|  |  |  |  |  |  |  |  |
| Continued |  |  |  |  |  |  |  |
| Code | RT | Compounds name by classes | Formula | CAS | RI | Peak area of volatile compounds | |
|  |  |  |  |  |  | RJ | LPFJ |
| A20 | 24.374 | 2-Nonen-1-ol | C9 H18 O | 22104-79-6 | 1406 | ND | 147221±1507 |
| A21 | 13.364 | 1-Butanol, 3-methyl- | C5 H12 O | 123-51-3 | 1215 | 1784108±258331 | 4742176±558196 |
| A |  | Total alcohols |  |  |  | 3993914±239405 | 12249013±714976 |
|  |  |  |  |  |  |  |  |
|  |  | Aldehydes |  |  |  |  |  |
| B1 | 19.441 | 2-Heptenal, (E)- | C7 H12 O | 18829-55-5 | 1324 | 312350±28523 | 8907426±781037 |
| B2 | 32.205 | Benzaldehyde | C7 H6 O | 100-52-7 | 1523 | 2164715±197251 | 1999024±168303 |
| B3 | 25.746 | 2-Octenal, (E)- | C8 H14 O | 2548-87-0 | 1428 | ND | 2759763±184921 |
| B4 | 7.535 | Hexanal | C6 H12 O | 66-25-1 | 1084 | 1924307±161994 | 117877±15294 |
| B5 | 17.514 | Octanal | C8 H16 O | 124-13-0 | 1305 | ND | 1034670±78821 |
| B6 | 19.939 | 2-Heptenal, (Z)- | C7 H12 O | 57266-86-1 | 1339 | ND | 936756±55229 |
| B7 | 42.027 | 2-Hexenal, 2-ethyl- | C8 H14 O | 645-62-5 | 1618 | ND | 610501±40346 |
| B8 | 65.359 | 5-Hydroxymethylfurfural | C6 H6 O3 | 67-47-0 | 2537 | 491517±40694 | ND |
| B9 | 28.794 | Furfural | C5 H4 O2 | 98-01-1 | 1473 | 278430±41223 | ND |
| B10 | 14.106 | 2,4-Nonadienal | C9 H14 O | 6750-03-4 | 1225 | ND | 236147±40072 |
| B11 | 33.172 | 2-Nonenal, (Z)- | C9 H16 O | 60784-31-8 | 1531 | ND | 207914±40031 |
| B12 | 51.563 | Benzaldehyde, 2,4-dimethyl- | C9 H10 O | 15764-16-6 | 1743 | ND | 156470±12090 |
| B13 | 64.092 | Butanal, 3-hydroxy- | C4 H8 O2 | 107-89-1 | 2307 | 17287±1063 | ND |
| B |  | Total aldehydes |  |  |  | 5188608±150368 | 16966551±1026500 |
|  |  |  |  |  |  |  |  |
|  |  | Esters |  |  |  |  |  |
| C1 | 42.458 | Decanoic acid, ethyl ester | C12 H24 O2 | 110-38-3 | 1625 | 3615908±122104 | 3132954±258161 |
|  |  |  |  |  |  |  |  |
| Continued |  |  |  |  |  |  |  |
| Code | RT | Compounds name by classes | Formula | CAS | RI | Peak area of volatile compounds | |
|  |  |  |  |  |  | RJ | LPFJ |
| C2 | 44.021 | Benzoic acid, ethyl ester | C9 H10 O2 | 93-89-0 | 1653 | 2918926±301498 | 1866498±233757 |
| C3 | 26.511 | Octanoic acid, ethyl ester | C10 H20 O2 | 106-32-1 | 1436 | 1826662±261866 | 1435721±106995 |
| C4 | 53.906 | Dodecanoic acid, ethyl ester | C14 H28 O2 | 106-33-2 | 1835 | 382539±42336 | 327267±16369 |
| C5 | 56.32 | 1-Butanol, 3-methyl-, benzoate | C12 H16 O2 | 94-46-2 | 1928 | 221939±6633 | 355419±38283 |
| C6 | 19.587 | Acetic acid, methyl ester | C3 H6 O2 | 79-20-9 | 1330 | 406582±45117 | ND |
| C7 | 61.512 | 2-Hydroxy-gamma-butyrolactone | C4 H6 O3 | 19444-84-9 | 2142 | 367058±12435 | ND |
| C8 | 14.496 | Hexanoic acid, ethyl ester | C8 H16 O2 | 123-66-0 | 1233 | 134018±11843 | ND |
| C9 | 15.763 | Hexanoic acid, 2-phenylethyl ester | C14 H20 O2 | 6290-37-5 | 1283 | ND | 146499±7298 |
| C10 | 55.466 | Benzenepropanoic acid, ethyl ester | C11 H14 O2 | 2021-28-5 | 1891 | 122009±16836 | ND |
| C11 | 52.414 | Acetic acid, 2-phenylethyl ester | C10 H12 O2 | 103-45-7 | 1811 | ND | 108294±9414 |
| C |  | Total esters |  |  |  | 9995644±685373 | 7372655±533211 |
|  |  |  |  |  |  |  |  |
|  |  | Acid |  |  |  |  |  |
| D1 | 28.685 | Acetic acid | C2 H4 O2 | 64-19-7 | 1460 | 786285±39893 | 14958413±769564 |
| D2 | 46.375 | Butanoic acid, 3-methyl- | C5 H10 O2 | 503-74-2 | 1686 | ND | 3149210±278850 |
| D3 | 54.753 | Hexanoic acid | C6 H12 O2 | 142-62-1 | 1843 | ND | 2830685±178764 |
| D4 | 59.975 | Octanoic acid | C8 H16 O2 | 124-07-2 | 2057 | 363133±11752 | 846158±8642 |
| D5 | 43.121 | Butanoic acid | C4 H8 O2 | 107-92-6 | 1629 | ND | 915166±55014 |
| D6 | 62.675 | n-Decanoic acid | C10 H20 O2 | 334-48-5 | 2263 | 551163±52691 | 275254±22993 |
| D7 | 61.445 | Nonanoic acid | C9 H18 O2 | 112-05-0 | 2138 | 82639±4637 | 357601±30961 |
| D8 | 37.989 | Propanoic acid, 2-methyl- | C4 H8 O2 | 79-31-2 | 1567 | ND | 284686±31310 |
| D9 | 58.034 | Heptanoic acid | C7 H14 O2 | 111-14-8 | 1978 | ND | 238227±36062 |
|  |  |  |  |  |  |  |  |
| Continued |  |  |  |  |  |  |  |
| Code | RT | Compounds name by classes | Formula | CAS | RI | Peak area of volatile compounds | |
|  |  |  |  |  |  | RJ | LPFJ |
| D10 | 49.861 | Propanedioic acid, propyl- | C6 H10 O4 | 616-62-6 | 1725 | ND | 104910±5335 |
| D11 | 64.834 | Benzoic acid | C7 H6 O2 | 65-85-0 | 2433 | 333244±30835 | ND |
| D |  | Total acids |  |  |  | 2116466±50179 | 23960315±1190659 |
|  |  |  |  |  |  |  |  |
|  |  | Ketones |  |  |  |  |  |
| E1 | 11.752 | 2-Heptanone | C7 H14 O | 110-43-0 | 1191 | ND | 16516781±1175153 |
| E2 | 17.473 | Acetoin | C4 H8 O2 | 513-86-0 | 1295 | ND | 7500342±727732 |
| E3 | 23.456 | 2-Nonanone | C9 H18 O | 821-55-6 | 1388 | ND | 6649409±562287 |
| E4 | 18.339 | 1-Octen-3-one | C8 H14 O | 4312-99-6 | 1315 | ND | 6610974±342673 |
| E5 | 38.563 | 2-Undecanone | C11 H22 O | 112-12-9 | 1598 | ND | 4193401±218086 |
| E6 | 2.234 | Acetone | C3 H6 O | 67-64-1 | 841 | 944508±186406 | ND |
| E7 | 20.505 | 5-Hepten-2-one, 6-methyl- | C8 H14 O | 110-93-0 | 1344 | 54382±8192 | 592579±54663 |
| E8 | 52.137 | 2-Tridecanone | C13 H26 O | 593-08-8 | 1807 | ND | 446474±39179 |
| E9 | 14.526 | 2-Heptanone, 6-methyl- | C8 H16 O | 928-68-7 | 1236 | ND | 85232±5426 |
| E10 | 15.403 | 3-Heptanone, 5-methyl- | C8 H16 O | 541-85-5 | 1265 | ND | 51202±4533 |
| E |  | Total ketones |  |  |  | 998890±178246 | 42646396±879305 |
|  |  |  |  |  |  |  |  |
|  |  | Others |  |  |  |  |  |
| F1 | 4.461 | Propane, 2-(ethenyloxy)- | C5 H10 O | 926-65-8 |  | ND | 7728534±923966 |
| F2 | 14.912 | 1,3,5,7-Cyclooctatetraene | C8 H8 | 629-20-9 | 1244 | 486777±57501 | ND |
| F3 | 59.016 | Phenol | C6 H6 O | 108-95-2 | 2023 | ND | 282782±43045 |
| F4 | 24.681 | 3-Nonyne | C9 H16 | 20184-89-8 | 1413 | ND | 71310±6344 |
|  |  |  |  |  |  |  |  |
| Continued |  |  |  |  |  |  |  |
| Code | RT | Compounds name by classes | Formula | CAS | RI | Peak area of volatile compounds | |
|  |  |  |  |  |  | RJ | LPFJ |
| F5 | 14.893 | Oxirane, hexyl- | C8 H16 O | 2984-50-1 | 1240 | ND | 36719±5062 |
| F6 | 9.567 | Oxirane, pentyl- | C7 H14 O | 5063-65-0 | 1153 | ND | 21154±1744 |
| F7 | 63.424 | DL-Arabinose | C5 H10 O5 | 20235-19-2 | 2281 | 17385±1568 | ND |
| F8 | 42.671 | (Z)-Undec-6-en-2-one | C11 H20 O | 107853-70-3 |  | ND | 1510754±111697 |
| F9 | 24.651 | 1,3-Hexadiene, 3-ethyl-2-methyl- | C9 H16 | 61142-36-7 | 1410 | ND | 113972±5280 |
| F10 | 14.38 | 1-(Hydroxymethyl)-1-(2'-hydroxyethyl) cyclopropane | C6 H12 O2 | 1000222-06-3 |  | ND | 93053±3486 |
| F11 | 59.216 | Carbonic acid, isohexyl phenyl ester | C13 H18 O3 | 1000314-57-0 | 2027 | 78218±3178 | ND |
| F |  | Total others |  |  |  | 582380±55352 | 9858283±807981 |

**ND, not detected.**
