# Supplementary figures and images for: Evaluating the effect of lactic acid bacteria fermentation on quality, aroma, and metabolites of chickpea milk
Source: Front Nutr. 2022 Dec 5;9:1069714. doi: 10.3389/fnut.2022.1069714 (PMC9760965; doi:10.3389/fnut.2022.1069714)

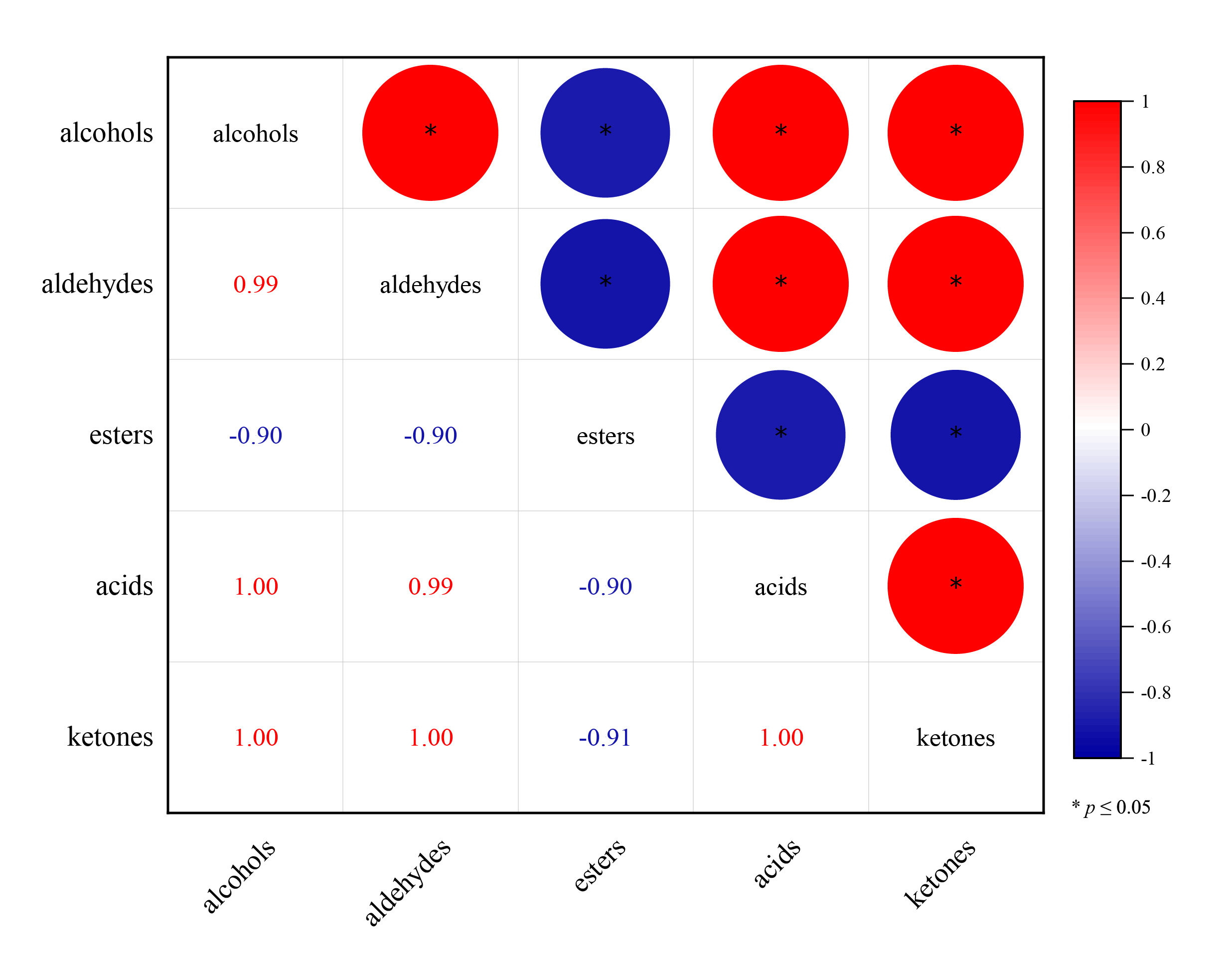

Supplement: Supplementary Figure 1 — Correlation between substances: red is positive correlation, blue is negative correlation; the size of the circle represents the degree of correlation (p < 0.05). [file Image_1.JPEG]

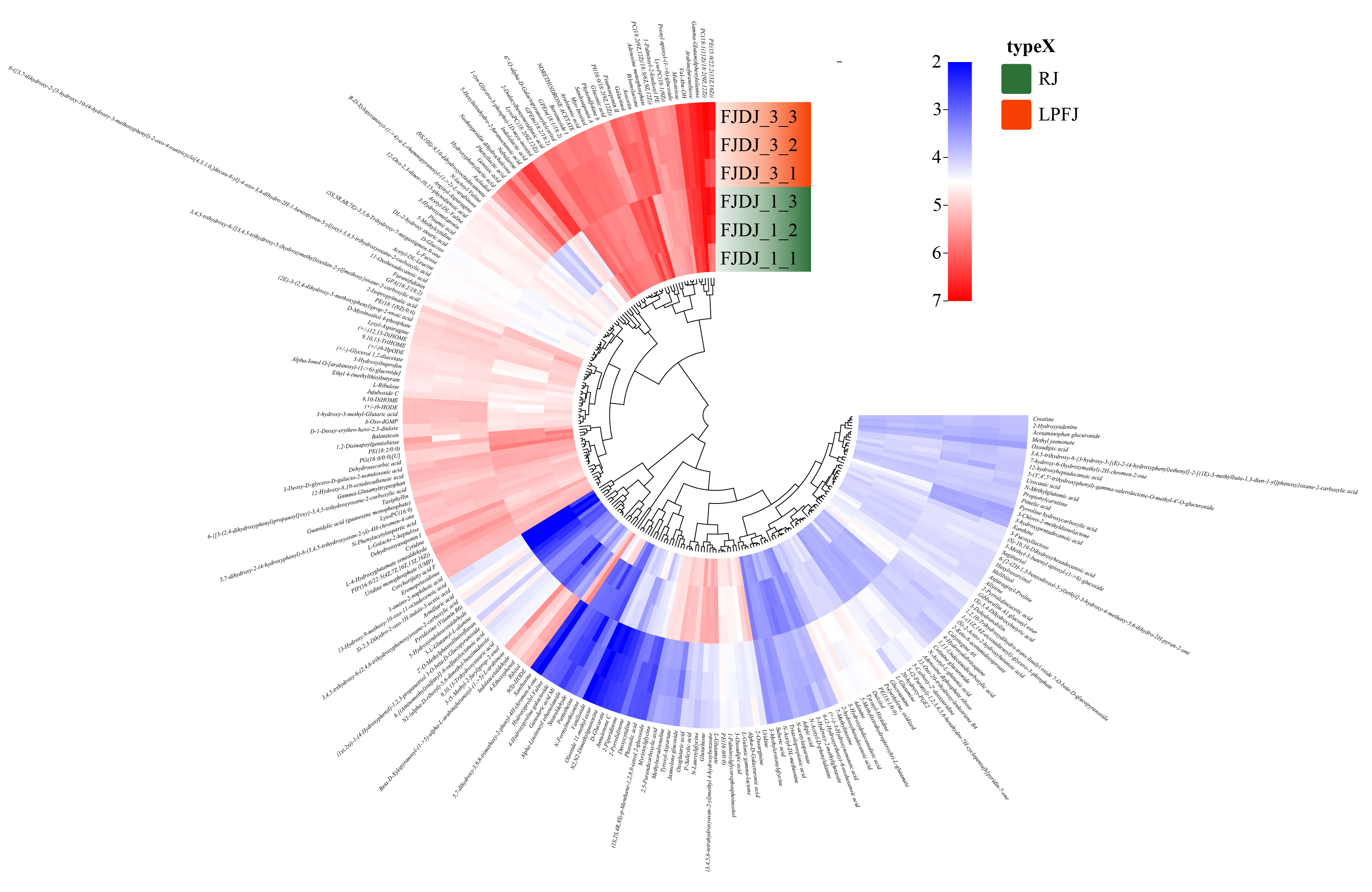

Supplement: Supplementary Figure 2 — Heatmap of hierarchical clustering analysis of differential abundant metabolites in RJ and LPFJ. [file Image_2.JPEG]

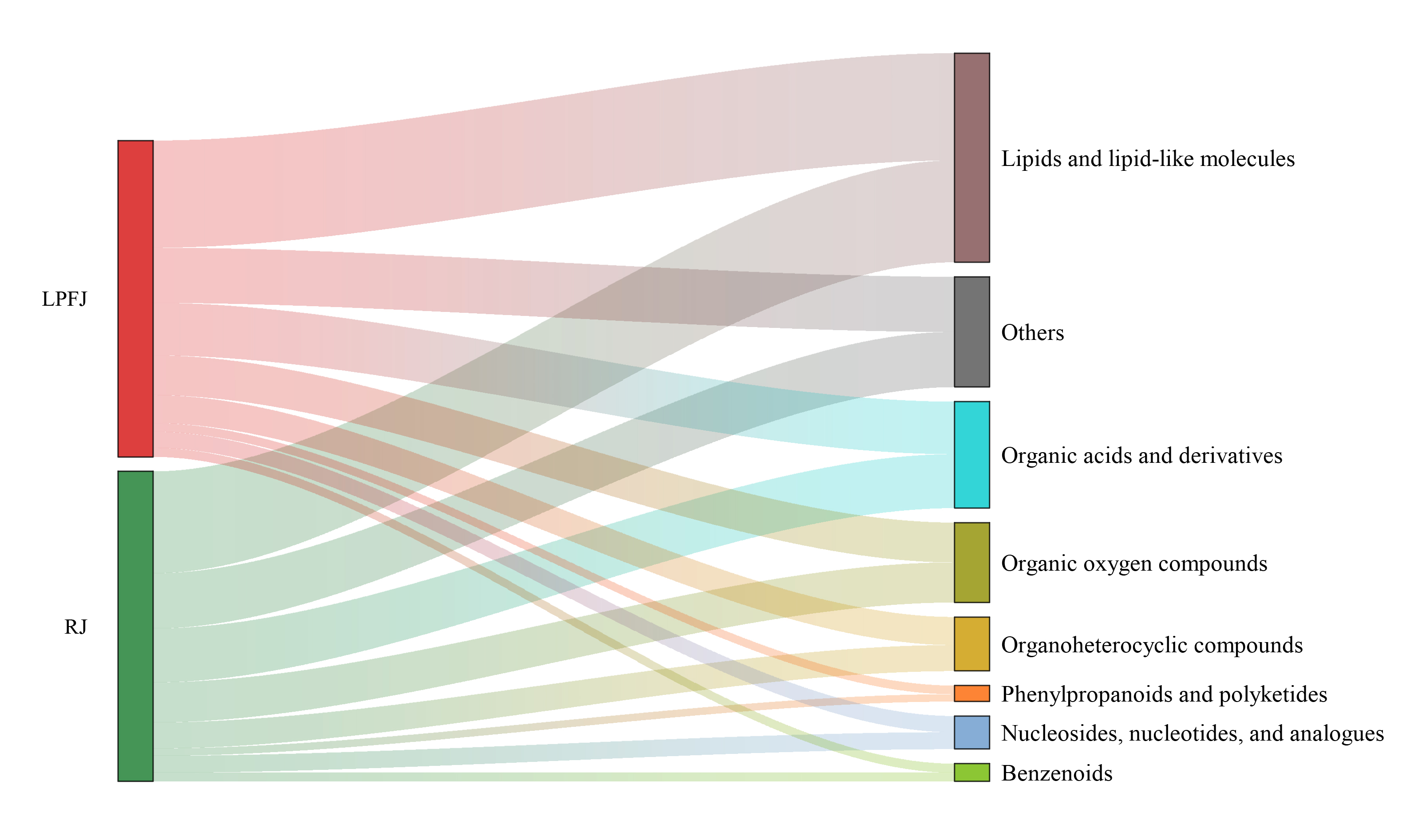

Supplement: Supplementary Figure 3 — Sankey plot of the changes of non-volatile metabolites in RJ and LPFJ based on HMDB super class. [file Image_3.JPEG]
